# Artificial intelligence guided acupuncture decision making and treatment: a review of research

**DOI:** 10.3389/fmed.2026.1752082

**Published:** 2026-04-27

**Authors:** Ru Nie, Kaiyue Deng, Jing He, Jialin Jia, Hongye Wang, Shuting Yuan, Tie Li

**Affiliations:** Department of Acupuncture and Tuina, Changchun University of Chinese Medicine, Changchun, Jilin, China

**Keywords:** acupuncture, artificial intelligence, clinical decision-making, review, treatment optimization

## Abstract

Artificial intelligence (AI) is increasingly applied in clinical acupuncture to modernize diagnosis and treatment. By addressing critical gaps in traditional practice, such as the lack of objective standardization in acupoint selection, reliance on subjective practitioner experience for localization, insufficient real-time safety monitoring, and the need for personalized efficacy prediction—AI offers significant clinical value. This paper reviews the application of AI in acupuncture across these four key areas. We summarize existing research and provide recommendations to guide the future development of intelligent acupuncture systems.

## 1 Introduction

Artificial intelligence has gradually penetrated the healthcare sector, opening up new avenues for development in medical research (1). In many developed countries and China, AI is rapidly advancing in healthcare, enabling intelligent medical data analysis and supporting disease diagnosis, treatment optimization, and personalized care (2). The potential for the development of Traditional Chinese Medicine with the assistance of artificial intelligence is immense (3, 4). As an important branch of Traditional Chinese Medicine, acupuncture has a history of over 3,000 years (5). Its therapeutic action lies in utilizing acupoints as reactive sites to disorders, and through stimulating these points, it treats various diseases and promotes rehabilitation (6, 7). Despite its extensive history and clinical benefits, traditional acupuncture faces several critical challenges in modern practice. These include the lack of objective standardization in acupoint selection, operator dependent subjectivity in anatomical localization, limited real-time safety monitoring during interventions, and an absence of individualized prognostic modeling for therapeutic outcomes. To address these limitations, there is a strong motivation to integrate artificial intelligence (AI) into clinical acupuncture. Existing reviews on the application of artificial intelligence in the field of Traditional Chinese Medicine are typically only broad overviews. Few studies directly reflect these technologies onto the clinical workflow of acupuncture. Therefore, the objectives of this paper are to systematically review the current state of AI applications in clinical acupuncture across four key areas: guiding acupoint selection, assisting localization, improving safety, and predicting efficacy. By summarizing existing research, objectively evaluate the challenges faced by AI in the clinical field of acupuncture, and provide suggestions for the combined development of artificial intelligence and traditional acupuncture theory.

## 2 Artificial intelligence methods in acupuncture data analysis

In the field of medical research, supervised learning, unsupervised learning, and deep learning are among the commonly used categories. In the analysis of acupuncture prescriptions, the selection of methods requires comprehensive consideration of data structure, annotation status, and clinical objectives. For structured data containing prescriptions and outcomes, when label quality is high and sample size is moderate, supervised learning algorithms such as support vector machines and tree-based ensemble models are relatively suitable, as they offer both good predictive performance and interpretability; however, they are sensitive to label noise and may perform poorly when dealing with sparse or imbalanced data (8). In contrast, unsupervised learning and association rule mining are applicable to the exploratory analysis of acupoint combinations, as they can identify co-occurrence patterns and core prescription structures without requiring outcome labels; however, the patterns obtained are correlational in nature and are easily influenced by parameter settings (9). Deep learning methods, on the other hand, offer advantages when processing high-dimensional or multimodal data such as electronic medical records and image-guided localization, as they support automated feature extraction and nonlinear modeling; however, they require large amounts of data and computational resources, and their interpretability is relatively limited (10). In general, supervised learning is suitable for outcome prediction and efficacy evaluation, association analysis focuses on mining prescription patterns, and deep learning performs best in multimodal integration scenarios. Method selection should strike a balance among accuracy, interpretability, data scale, and clinical applicability to ensure the robustness and practical utility of the analytical results.

## 3 Guided decision making and treatment

### 3.1 Assisted acupoint selection

Acupoints are specific sites where the physiological activity of internal organs and meridians manifests on the body surface. They reflect diseases and serve as stimulation targets in acupuncture, playing a key role in diagnosis and treatment (11). When the body is diseased, acupoints may show tenderness or nodules. Stimulating these points unblocks meridians, regulates Qi and blood, and restores balance to achieve therapeutic effects (12). AI assists acupuncturists in selecting optimal points by analyzing clinical efficacy data (13). Accurate acupoint selection is crucial for clinical outcomes, forming the basis of an effective prescription. Recent advances in medical informatics and big data have driven progress in medical data mining and knowledge discovery (14). Among these, data mining methods have been applied in acupuncture, particularly in the evaluation of acupuncture’s effectiveness in treating various diseases (15–21). Traditionally, acupoint selection relies heavily on subjective experience, leading to inconsistent treatments. AI data mining reveals acupoint patterns and efficacy correlations from clinical and literature data, supporting standardized prescriptions. Common methods include.

#### 3.1.1 Association rule mining

**Figure 1:**
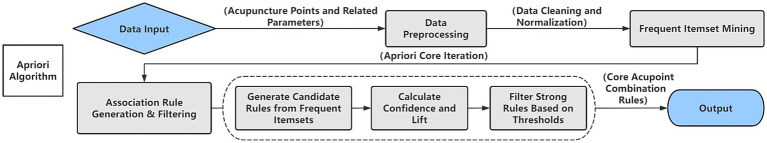
Flowchart of the Apriori algorithm.

Proposed by Rakesh Agrawal and Ramakrishnan Srikant in 1994, the Apriori algorithm remains a foundational technique for association rule mining within large datasets. It operates through a two-stage process: first, identifying frequent itemsets that exceed a predefined minimum support threshold, and subsequently extracting association rules from these itemsets that satisfy a minimum confidence threshold (22). Support and confidence are two key metrics used to measure the strength of associations in data mining. Support represents the frequency with which both the antecedent and consequent appear together in the dataset, while confidence indicates the likelihood that the consequent will appear when the antecedent is present. Association rules that satisfy both the minimum support and minimum confidence thresholds are referred to as strong association rules. The Apriori algorithm holds significant value in the exploration of acupoint combination patterns in acupuncture. By analyzing large volumes of clinical case data, it can automatically identify frequently co-occurring acupoint combinations and their association rules with specific diseases. Additionally, the Apriori algorithm can identify multi-core acupoint clusters and uncover potential patterns of synergistic acupoint combinations. Researchers have validated many classic acupoint combination patterns through empirical studies. For example, the algorithm can uncover frequently used acupoints in the treatment of gastric pain, such as Zhongwan (CV12), Shangwan (CV13), and Zusanli (ST36). It can also reveal associations between factors like etiology and pathogenesis with acupoint selection, the correlation between comorbid conditions and acupoint selection, as well as the relationship between acupoint choice and acupuncture techniques (23). For instance, Fengchi (GB20) combined with Jianjiasi (the cervical paravertebral points), and Houxi (SI3) with Jianjiasi represent typical acupoint combinations in the treatment of neurogenic cervical spondylosis (24). Sanyinjiao (SP6), Qihai (CV6), and Guanyuan (CV4) are among the most commonly used acupoints for treating dysmenorrhea (painful menstruation) (25). This association rule-based analytical approach has validated the scientific basis of traditional acupoint combination theories. The specific procedure is detailed in Figure 1.

#### 3.1.2 Clustering analysis

**Figure 2:**
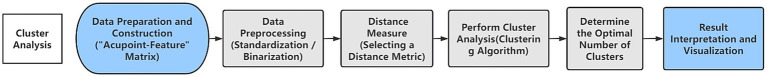
Flowchart of clustering analysis.

Clustering analysis is an important unsupervised learning method in artificial intelligence. It automatically groups acupoints that frequently appear together in clinical prescriptions, forming distinct clusters. These clusters can be interpreted as representing different treatment strategies for a specific disease, acupoint combination approaches, or the idiosyncratic methodologies of specific traditional acupuncture lineages. Empirical applications of this technique have yielded significant clinical insights. For instance, in the management of stable chronic obstructive pulmonary disease (COPD) (26), clustering algorithms were utilized to partition frequently employed acupoints into five functional groups. The primary core cluster comprised Shenshu (BL23), Pishu (BL20), Feishu (BL13), and Taiyuan (LU9). Similarly, in the context of chronic heart failure (26), clustering analysis categorized commonly prescribed acupoints into two primary therapeutic clusters, with the dominant group featuring Neiguan (PC6), Xinshu (BL15), and Danzhong (CV17). This approach has also been applied in many other studies (15, 19, 27). These core acupoint groups provide data-driven, standardized protocols that reduce clinical subjectivity and improve treatment consistency. The specific procedure is detailed in Figure 2.

In addition to the two main methods mentioned above, artificial neural networks (ANNs), as a key component of artificial intelligence, possess powerful learning capabilities and the ability to process complex data. By learning from prototype building data, artificial neural networks (ANNs) can support passive design and form optimization, and have also been applied to guide acupoint selection in acupuncture. For instance, a study (28) validated the feasibility of using artificial neural networks (ANNs) in acupuncture by collecting 232 clinical case records. The researchers trained the artificial neural networks (ANNs) model to analyze the relationship between 87 symptoms and 77 commonly used acupoints, predicting acupoint selection with 0.865 accuracy. This shows artificial neural networks (ANNs) can guide clinical decisions using prior data. Other teams have also explored intelligent acupoint selection. Liu et al. (29) proposed a graph theory-based method for intelligent acupoint selection in acupuncture robots. By constructing an acupoint network from Traditional Chinese Medicine classics and applying a community detection algorithm, they identified symptom-specific acupoint clusters, providing a theoretical basis for robotic acupuncture. To address the low accuracy and limited applicability of existing core acupoint mining algorithms, Zhao et al. (30) developed a core acupoint mining algorithm using 3-node motifs in an acupoint-disease network. By reconstructing edge weights and applying an improved PageRank algorithm, they identified core acupoints with higher resolution and accuracy than conventional methods.

#### 3.1.3 Summary

AI-driven data mining techniques like association rule mining and clustering analysis provide objective data support for standardized acupoint selection. However, there is still room for further development in these techniques. The limitation of the Apriori algorithm lies in its reliance on statistical correlation rather than causal inference. As a common association rule mining tool, the Apriori algorithm efficiently identifies frequently co-occurring acupoint combinations. However, the rules it generates reflect only statistical correlations, not causal relationships or therapeutic mechanisms, and many lack clinical significance. The algorithm also requires high-quality data; irregularities in medical records may produce misleading rules. Therefore, Apriori is best suited for generating data hypotheses that require further validation before clinical application. A key limitation of clustering analysis is its potential to produce meaningless groupings. While it automatically clusters acupoints based on data structure, interpretation of these clusters relies entirely on researcher subjectivity, making results susceptible to bias. For the Apriori algorithm, future research should integrate it with other techniques to assess whether acupoint combinations contribute to therapeutic efficacy. For clustering analysis, future research should focus on incorporating Traditional Chinese Medicine knowledge into the clustering process. For example, the clustering analysis could group acupoints such as Zusanli (ST36), Pishu (BL20), and Zhongwan (CV12) into a functional module related to strengthening the spleen and stomach.

### 3.2 Assisted acupoint localization

Acupoints, identified through anatomical landmarks, palpation, and clinical experience, are invisible to the naked eye (31). Hundreds of acupoints exist along meridians, each associated with specific therapeutic effects. For instance, needling Neiguan (PC6) prevents nausea (32), and needling Zusanli (ST36) improves gastrointestinal disorders (33). Accurate localization is the fundamental prerequisite for achieving these therapeutic effects and ensuring patient safety, as even minor spatial deviations can significantly compromise clinical outcomes or increase the risk of adverse events. Traditionally, acupoint location relies on surface landmarks, bone measurements, and finger-based *cun* measurement, along with palpation. However, individual differences in body size cause variations in *cun* measurement and point positioning, affecting localization accuracy. Additionally, the practitioner’s subjective factors can further influence precision (34, 35). To overcome these limitations, establishing an objective and standardized localization framework is crucial. The rapid development of AI has provided new methods for assisting acupoint localization in acupuncture. The following sections review the current state of research regarding AI-assisted acupoint localization.

#### 3.2.1 Location of points on the head and face

A predominant approach in facial acupoint localization uses deep learning to detect anatomical landmarks, and then infers acupoint coordinates based on spatial relationships to these keypoints. For instance, Chang (36) deployed an Active Shape Model (ASM) to identify 83 facial feature points, integrating Canny edge detection with proportional bone measurement (*cun*) to derive acupoint coordinates. A backpropagation (BP) neural network was subsequently introduced to predict the locations of acupoints devoid of distinct visual features. Liu et al. (37) proposed a lightweight Faster PFLD network to detect 98 facial landmarks, facilitating the localization of 16 specific acupoints. Comparable methodologies were adopted by Zheng (38) and Zhang (39), who utilized an optimized PFLD and a 468-point 3D facial mesh generated via MediaPipe. By synthesizing these meshes with proportional *cun* ratios, these models achieved precise positioning. The maturation of these technologies has also led to the emergence of numerous interactive applications. For example, Lan et al. (40) and Su et al. (41) developed augmented reality (AR)-based smartphone applications that can display acupoint locations in real-time on the user’s face. Zheng (38) further developed a mobile system using Unity3D that integrates querying, recommendations, and AR visualization. Chen et al. (42) expanded this technology to mixed reality, implementing 3D real-time acupoint recognition and visualization on the HoloLens 2.

To avoid intermediate errors, many studies use direct localization methods, employing deep learning models to regress acupoint coordinates straight from images. For instance, Yuan et al. (43) proposed an improved YOLOv8-pose model, which can directly detect 11 facial acupoints. Chen et al. (44, 45) utilized a deep network based on ResNet50, employing transfer learning and an adaptive Wing Loss function to enhance the localization accuracy. Gao (46) introduced generative adversarial networks (GANs) into this field, transforming the acupoint localization problem into an image generation task. By constructing a paired facial-acupoint image dataset and designing an acupoint map estimation network with an improved cycle-consistent generative adversarial network (CycleGAN) structure, this approach achieved high-precision eye acupoint localization even with limited data. To further enhance performance, researchers have turned to high-resolution networks (HRNet), which are better at preserving spatial details. Li (47) innovatively utilized RGB-D (red, green, blue, and depth) multimodal data to create the first RGB-D (red, green, blue, and depth) facial dataset with acupoint annotations. He also designed a dual-branch high-resolution network (DA-HRNet). Zhang et al. (48, 49) created the FAcupoint dataset covering 43 acupoints. They developed a detection method combining high-resolution networks (HRNet) with an attention fusion module (RCSAF), and later proposed a self-supervised learning framework that learns robust facial features from unlabeled data, achieving very low normalized mean error in small sample scenarios. For ear acupoint localization, Wang et al. (50) developed “YoloEar21,” an AI system using the YOLOv11 pose estimation model to simultaneously detect ear positioning and 21 acupoint key points. The model was deployed as a cross-platform web application.

In addition, some studies have focused on building expert knowledge-based systems to guide acupoint localization. Yang et al. (51) and Ye et al. (52) both proposed integrating clinical localization data from multiple experts to calculate the optimal precision value for acupoints. Based on this, they developed virtual systems for learning and automating acupoint localization. The specific details are presented in Table 1.

**Table 1:** Location of points on the head and face.

Region	Author	Core method	Evaluation metric
Face	Zhang et al. (39)	MediaPipe	NME = 3.9%，FR = 5.13%
Face	Yuan et al. (43)	YOLOv8-ACU	mAP@0.5 = 97.5% mAP@0.5:0.95 = 76.9% Precision = 92.2% Recall = 97.0%
Face	Liu et al. (37)	Faster PFLD	MSE = 2.2128; NME = 5.20%
Face	Li (47)	DA-HRNet、ADCA-HRNet	NME of the two models: DA-HRNet = 5.42% ADCA-HRNet = 4.85%
Face	Yang et al. (44)	Haar cascade classifier, AdaBoost	______
Face	Lan et al. (40)	3D Morphable Model	Error = 2.4 mm
Face	Chen et al. (44)	ResNet50	NME = 2.5%
Face	Zheng (38)	Improved MTCNN + improved PFLD	Accuracy >94%
Face	Chen (45)	ResNet-50 + transfer learning, ALHRNet	NME = 2.50% and NME = 1.76%
Face	Chen et al. (42)	Dlib, 3DMM/SFM	Absolute error = 2.55 mm, Relative error = 1.18%
Face	Chang (36)	BP neural network	Error = 5.44 pixels
Face	Ye et al. (52)	A custom information fusion algorithm model	______
Face	Zhang et al. (48)	HRNet、RCSfNet、FADbR	FA_NME = 1.2866%
Face	Su et al. (41)	Google ML Kit	NME = 3.376%、FR = 4.348%
Face	Zhang et al. (49)	FADbR	NME = 1.8632% FR = 0% AUC = 0.8070
Ear	Wang et al. (50)	YOLOv11x	mAP = 0.982–0.983, Precision = 0.975–0.991, Recall = 0.976
Eye	Gao (46)	Improved CycleGAN	OAR = 75.11%, ACE = 5.245

NME, Normalized Mean Error; FR, Failure Rate; mAP, Mean Average Precision; MSE, Mean Squared Error; AUC, Area Under the Curve; OAR, Overlap Area Ratio; ACE, Average Center Error; PFLD, Practical Facial Landmark Detector; HRNet, High-Resolution Network; MTCNN, Multi-task Cascaded Convolutional Networks; ResNet, Residual Network; 3DMM, 3D Morphable Model; SFM, Structure from Motion; BP, Back Propagation; GAN, Generative Adversarial Network.

#### 3.2.2 Location of points on the hand

Hand acupoint localization predominantly employs indirect strategies, utilizing frameworks like MediaPipe or OpenPose to detect skeletal landmarks, from which acupoint coordinates are geometrically inferred. For instance, Lee et al. (53) applied OpenPose with geometric transformations to approximate dorsal acupoints like Shaoze (SI1) and Hegu (LI4). Tian et al. (54) validated this approach in Mongolian medicine, utilizing MediaPipe keypoints with arithmetic averaging and polynomial fitting to achieve clinically viable error margins (< 1 cm). Similarly, Wang et al. (55) integrated high-resolution networks (HRNet) heatmap regression with traditional *cun* measurements via a cascaded architecture. To address morphological heterogeneity, Chen (56) stratified hand acupoints into four tailored strategies: (1) direct coordinate offsetting from MediaPipe keypoints; (2) integrating keypoints with Canny-extracted contours; (3) utilizing a YOLOv8s nail-detection reference for peri-ungual acupoints; and (4) regressing coordinates directly from 21 keypoints via a fully connected neural network. This multi-strategy paradigm significantly enhances localization robustness.

Building on indirect localization, some studies integrate Traditional Chinese Medicine functional zone information with key point topological relationships to guide models for more accurate acupoint localization. Chen (57) and Zheng et al. (58) developed a three-step acupoint localization method: first, an improved DeepLabV3 segments the palm into 14 functional zones; second, MediaPipe detects 21 skeletal key points to construct a topological relationship map; third, these maps are fused and input into YOLOv8 to regress 16 acupoint coordinates. By directly embedding Traditional Chinese Medicine functional zone and anatomical relationship information, this fusion method narrows the model’s search space, improving localization accuracy and alignment with meridian principles.

Direct localization methods regress heatmaps from input images to identify acupoint coordinates, primarily focusing on architectural and loss function optimization. For instance, Cao et al. (59) augmented the High-Resolution Network (HRNet) with Spatial and Channel Reconstruction Convolution (SCConv) and a feature decoupling module to mitigate redundancy, utilizing a mutual information loss to enhance feature relevance. Alternatively, Chang et al. (60) proposed a coarse-to-fine “meridian-to-acupoint” paradigm. By integrating U-Net for hand segmentation with an attention-augmented ResNet and an online hard keypoint mining mean squared error (MSE) loss, this approach leverages prominent meridian features as strong spatial constraints for precise acupoint regression.

In addition to the methods mentioned above, many other innovative approaches have been proposed. One such approach reframes the problem as an image registration task. For example, Li et al. (61) introduced Acupoint Image Registration Network (AIR-Net), which computes spatial transformations between test images and a pre-labeled reference atlas to map coordinates. This approach exhibits robust few-shot learning capabilities, achieving cross-individual registration with minimal training data. Incorporating 3D vision, Masood (62) utilized RGB-D (red, green, blue, and depth) data and a dual-stream Convolutional Neural Network (CNN) to fuse color and depth features for 3D spatial coordinate prediction. Furthermore, for real-time deployment on edge devices, Wang et al. (63) proposed LECA—a lightweight network using MobileNetV2, an efficient channel attention module, and Huber loss. With only 1.67 M parameters, it maintains high accuracy. Detailed methodology comparisons are in Table 2.

**Table 2:** Location of points on the hand.

Region	Author	Core method	Ealuation metric
Hand	Li et al. (61)	AIR-Net	AARE = 2.57 PCK@0.05 = 0.73 PCK@0.10 = 0.94 PCK@0.15 = 0.98
Hand	Chen (57)	DeeplabV3, MediaPipe, YOLOv8	Error = 1.737 mm
Hand	Cao et al. (59)	HRNet	AP = 74.2% AP@50 = 91.2% AP@75 = 81.4% PCK = 94.2% NME = 0.06
Hand	Masood et al. (62)	RGB-D dual-stream CNN fusion, MediaPipe	MNE < 0.09
Hand	Chen (56)	MediaPipe, YOLOv8s, FCNN	MPE = 9.96 pixels and MPE = 14.8 pixels
Hand	Tian et al. (54)	YOLOv3, MediaPipe	Normalized offset < 0.083
Hand	Wang et al. (63)	LECA-MobileNetV2	AP 50 = 87.7% AR 50 = 88.1% Param = 1.67 M
Hand	Chang et al. (60)	ResNet152, U-Net, SE-Attention	Accuracy = 92.2%
Hand	Wang et al. (55)	SC-YOLOv5, HRNet	AOE = 0.0269
Hand	Zheng et al. (58)	Improved DeepLabV3, MediaPipe, YOLOv8	Error = 1.737 mm
Hand	Lee et al. (53)	OpenPose	Error ≈ 10 pixels

AARE, Average Absolute Relative Error; PCK, Percentage of Correct Keypoints; AP, Average Precision; AR, Average Recall; NME, Normalized Mean Error; MPE, Mean Pixel Error; AOE, Average Orientation Error.

#### 3.2.3 Location of points on the back

The back is a key region in Traditional Chinese Medicine, containing the Du Mai and Bladder meridian acupoints effective for various conditions. Current AI localization strategies mainly fall into indirect approaches, detecting anatomical keypoints followed by proportional measurement, and direct methods.

In indirect localization, which involves detecting key points first, Kong et al. (64) utilized high-resolution networks (HRNet) to detect shoulder keypoints and derive the spinal midline, subsequently calculating acupoint coordinates via the proportional bone measurement (*cun*) method. Similarly, Fu et al. (65) identified 18 skeletal keypoints to serve as geometric references, while Zhang et al. (66) integrated Faster R-CNN for bounding box detection with high-resolution networks (HRNet) for keypoint heatmap regression, establishing a pixel-to-*cun* mapping to compute relative anatomical positioning. To address clothing occlusion and morphological variations, Cao et al. (67) categorized back types into narrow, standard, and wide. Using a multilayer perceptron (MLP) trained on categorical data, they set Dazhui (GV14) as the coordinate origin and derived 59 acupoints via the *cun* method, enhancing robustness against variations in body habitus, posture, and muscle mass.

Alternatively, hierarchical indirect localization strategies progressively constrain the search space to enhance precision. Chang (68) proposed a coarse-to-fine paradigm utilizing an optimized Faster R-CNN to crop the region of interest (ROI), effectively mitigating background noise. Subsequently, CNN-based heatmap regression and Transformer-based SimC models were applied to the region of interest (ROI) for precise localization. A parallel two-stage framework was developed by Liu et al. (69), who employed an enhanced Keypoint R-CNN to generate initial acupoint proposals, followed by a knowledge-driven post-processing algorithm for refinement.

In direct localization, Zhang et al. (70) developed a streamlined variant of the OpenPose architecture, eliminating the need for structural priors to directly regress heatmaps for three specific back acupoints. More recently, Yang et al. (71) introduced AL-WFBS, a novel Transformer-based architecture capable of directly generating acupoint heatmaps, demonstrating the efficacy of self-attention mechanisms in this domain. Detailed methodological comparisons are summarized in Table 3.

**Table 3:** Location of points on the back.

Region	Author	Core method	Evaluation metric
Back	Chang (68)	Improved Faster R-CNN, CNRN-Heatmap, CPVT-SimCC	Accuracy = 94%
Back	Cao et al. (67)	AlphaPose, 5-LPM	Accuracy = 94.87% (Dazhui), 91.58% (Others)
Back	Zhang et al. (70)	Improved OpenPose	Detection rate ≈ 74.9%
Back	Kong et al. (64)	Faster R-CNN、HRNet	Error ≈ 5.3 mm
Back	Fu et al. (65)	VGG-19， A multi-stage convolutional neural network	Stereo/TOF error ≤ clinical threshold (≤25° rotation)
Back	Liu et al. (69)	Improved Keypoint R-CNN (ResNet-50 + FPN)	Accuracy = 90.12%
Back	Yang et al. (71)	AL-WFBS network (ResNet-50 + ResNet-50 + Transformer, etc.)	AAPE = 9.29, PCK@0.05 = 0.93, PCK@0.10 = 1.0
Back	Zhang et al. (66)	Faster R-CNN， HRNet	Detection rate > 99%, Error < 0.05 *cun*

PCK, Percentage of Correct Keypoints; AAPE, Average Absolute Position Error; FPN, Feature Pyramid Network; CNRN, Convolutional Neural Regression Network; cun, A traditional Chinese unit of length used in acupuncture.

#### 3.2.4 Location of points on the abdomen

The abdomen contains numerous meridian acupoints. Apart from Shenque (CV8), most lack clear surface landmarks, unlike facial points. This makes direct detection impossible, requiring indirect topological inference for localization.

Zhang et al. (72) developed a CNN-based system for automatic abdominal acupoint localization. Using a multi-task architecture, it detects Shenque (CV8) and segments the body boundary, then infers four key points: Shangwan (CV13), Qugu (CV2), and bilateral Daheng (SP15). Empirical evaluations demonstrated the system achieved a localization accuracy of 97.9% on 40 abdominal images. The specific details are presented in Table 4.

**Table 4:** Location of points on the abdomen.

Region	Author	Core method	Evaluation metric
Abdomen	Zhang et al. (72)	HigherHRNet	Accuracy = 97.9%

HRNet, High-Resolution Network.

#### 3.2.5 Multi-site localization

For multi-regional acupoint localization, a study (73) proposed two methods: first, using MediaPipe to detect facial and hand landmarks and derive 38 acupoints via proportional measurement; second, employing YOLOv8-pose to directly regress 5 arm acupoints. Both achieve high precision and real-time performance. For back and leg localization, Hu et al. (74) developed a method for Traditional Chinese Medicine massage robots using Symmetric Spatial Transformer Network (SSTN) based pose estimation to capture joint coordinates, proportional measurement to calculate 2D acupoint coordinates, and coordinate registration for 3D spatial mapping. The approach adapts well to body variations, clothing occlusion, and posture changes. Details are in Table 5.

**Table 5:** Multi-site localization.

Region	Author	Core method	Evaluation metric
Face, hand, and arm	Malekroodi et al. (73)	MediaPipe, YOLOv8-pose	Error ≈ 5.58 pixels
Back and leg	Hu (74)	SSTN, STN, SDTN, Hourglassk	Error = 23.615 mm

mAP, Mean Average Precision; OKS, Object Keypoint Similarity; SSTN, Spatial-Sequential Transformer Network; STN, Spatial Transformer Network.

#### 3.2.6 Summary

Acupoint localization falls into two main strategies: indirect and direct. Indirect methods such as MediaPipe and OpenPose detect skeletal keypoints and infer acupoint coordinates via geometric relationships, offering low technical barriers and high efficiency, but accuracy depends on keypoint quality and is prone to error accumulation. Direct methods including HRNet, YOLO, and Transformer avoid intermediate errors but rely heavily on dataset quality, with high-quality dataset construction remaining a challenge. Despite high theoretical accuracy, the clinical value of AI localization models requires comparison with traditional manual techniques. Experienced acupuncturists rely on dynamic palpation to perceive *Deqi*, a process currently irreplaceable by visual technologies. Thus, AI’s clinical significance lies in providing objective coordinates and supporting telemedicine, rather than replacing senior practitioners. Clinical acceptance remains a primary challenge. While AI computes coordinates rapidly, preparations such as equipment calibration, headset donning, and patient immobilization disrupt clinical workflows and impair doctor-patient interaction. Furthermore, algorithmic stability in real-world clinical environments lacks validation. Existing studies primarily involve standard somatotypes under controlled conditions. In practice, patients present dynamic postures, diverse body types, scoliosis, and clothing occlusion. Algorithms reliant on fixed anthropometric standards or skeletal keypoints often fail against these individual discrepancies. Future research must transition from 2D visual localization to multimodal fusion, integrating 3D spatial detection with tactile sensing. Moreover, large-sample clinical trials are essential to verify practical accuracy and efficiency.

### 3.3 Improving acupuncture safety

#### 3.3.1 Needle management safety

Needle management in acupuncture carries safety risks, as breakage or retention may result in severe complications such as organ perforation or pneumothorax (75, 76). At present, clinics and hospitals commonly use paper forms for manually recording the number of needles used and recovered. This method is not only time-consuming but also prone to errors. Patients being inadvertently discharged with retained needles. To prevent these errors and improve patient safety, researchers are now using AI to automate the tracking process.

Cheng et al. (77) developed a camera-based system that automatically counts and reconciles the number of inserted and recovered needles. In hospital trials, the system successfully achieved a zero retention rate, demonstrating its clinical feasibility. However, its identification accuracy decreased during high-density insertions due to visual occlusion in the images. Addressing this spatial limitation, Huang et al. (78) introduced AcuCount, a cloud-based needle counting algorithm utilizing a deep learning Oriented Region-based Convolutional Neural Network (R-CNN) model. By employing an optimized oriented object detection approach, the system effectively processes complex overlapping scenarios. In tests involving 20 needles, it achieved an accuracy of 96.49%, a precision of 99.98%, and a recall of 99.84%. Furthermore, to dynamically tackle both needle breakage and retention, Lin et al. (79) designed a real-time monitoring system based on the YOLOv8 architecture. Trained on clinical and public library images, the system provides high-precision detection of subcutaneous needles. With an average precision of 88.0% and a recall rate of 82.9%, this automated system effectively enhances procedural safety and treatment efficacy.

#### 3.3.2 Treatment procedure safety

Beyond algorithmic safety, the clinical execution safety of AI-driven robotic interventions has also been empirically evaluated. Zhang et al. (80) developed an intelligent moxibustion robot that autonomously regulates peri-acupoint skin temperature. In an randomized controlled trial (RCT) for primary dysmenorrhea, both robot and manual groups showed significant symptom improvement after three cycles (*p* < 0.05), with no significant efficacy difference between groups (*p* > 0.05). However, the robot group had significantly fewer adverse events (2.1% vs. 7.2%, *p* < 0.05), demonstrating non-inferior efficacy with superior safety. Further large-scale trials are needed to validate long-term outcomes.

#### 3.3.3 Summary

Acupuncture is increasingly recognized as a safe non-pharmacological therapy, with a very low incidence of severe adverse events under professional operation. However, in countries where it is practiced, some adverse events are inevitable (81). Integrating AI into acupuncture safety is a logical next step, but research remains in early stages. Existing needle management systems require validation through large-scale real-world studies. Key challenges include clinical accuracy, ease of use, and seamless integration into workflows without added burden. Further research is needed to confirm safety and cost-effectiveness.

### 3.4 Predicting therapeutic efficacy of acupuncture

Machine learning, as a flexible tool for handling complex medical data, has been used in predicting clinical therapeutic outcomes and decision-making (82, 83). Accurate efficacy prediction is of critical clinical importance in acupuncture treatment, as individual patient’s exhibit substantial variability in their therapeutic responses. By identifying patients who are likely to respond to acupuncture, as well as those who may not derive significant benefit prior to intervention, clinicians can avoid unnecessary utilization of medical resources, prevent delays in seeking alternative therapies, and formulate optimal personalized treatment plans to achieve better therapeutic outcomes. Current research on acupuncture efficacy prediction primarily focuses on two aspects. First, optimizing efficacy evaluation systems using existing clinical and imaging data. This utilizes artificial intelligence to enhance the objectivity and consistency of efficacy assessments, encompassing efficacy classification, score change prediction, and efficacy stratification. Second, constructing pre-treatment predictive models to estimate individualized treatment response probabilities prior to intervention, thereby improving clinical decision-making and resource allocation efficiency. The former emphasizes analyzing existing efficacy outcomes, whereas the latter focuses on prospective prediction and individualized risk stratification. Based on this dual-dimensional analytical framework, this study further categorizes the discussion by data source.

#### 3.4.1 Neuroimaging feature prediction

##### 3.4.1.1 Prediction based on spontaneous activity patterns of local brain regions

Indicators such as amplitude of low-frequency fluctuation (ALFF) or its fractional form (fALFF) are commonly used to measure the strength of spontaneous neural activity in local brain regions (84). Studies show acupuncture alters these values, improving neuronal dysfunction (85, 86). In migraine without aura, pre-treatment zALFF in bilateral occipital lobes predicted headache improvement, explaining 38 and 28% of variance (87). For neck pain, Gao et al. (88) used an support vector machine (SVM) model, finding pre-treatment amplitude of low-frequency fluctuation (ALFF) features in regions like the right middle temporal gyrus achieved 82.5% accuracy in distinguishing responders. In chronic sciatica, dynamic fALFF changes in parietal regions correlated with analgesic effects, with baseline values predicting functional improvement (89).

##### 3.4.1.2 Prediction based on brain network functional connectivity

Research has found that the human brain is a highly complex system composed of multiple specific functional networks (90). In brain network research, functional connectivity refers to the temporal synchronization of activity between different brain regions, reflecting the brain’s information exchange and functional organization, and serving as an important basis for disease prediction (91–94). In acupuncture research, functional connectivity patterns between brain networks have been shown to effectively predict therapeutic outcomes for various diseases. A chronic low back pain study (95) found baseline functional connectivity between the medial prefrontal cortex and regions like the insula predicted real vs. sham acupuncture efficacy, explaining 34 and 29% of variance, suggesting different neural pathways. Yu et al. (96) showed baseline connectivity patterns across pain-related brain networks predicted analgesic effects in dysmenorrhea, indicating acupuncture works by reconstructing network balance. For functional dyspepsia, Yin et al. (97) achieved 76% accuracy in predicting responders using reward circuit connectivity features, linking acupuncture’s effects to motivation and emotion processing systems.

##### 3.4.1.3 Other prediction methods

Liu et al. (98) integrated clinical features and resting-state functional magnetic resonance imaging (fMRI) data from migraine patients. Using partial least squares correlation analysis and hierarchical clustering, they identified two subgroups with distinct biological characteristics, including differences in headache frequency, depression scores, and functional connectivity patterns involving the amygdala, hippocampus, and thalamus. After electroacupuncture treatment, the two subgroups showed markedly different clinical response rates: 18% in Subgroup 1 versus 44% in Subgroup 2.

In brain structural feature prediction, Yang et al. (99) used a linear support vector machine (SVM) model and found that pre-treatment gray matter volume in 10 brain regions predicted acupuncture response in migraine without aura with 83% accuracy, suggesting structural biomarkers. For imaging features, Tang et al. (100) applied deep learning object detection models to analyze digital subtraction angiography (DSA) images from ischemic stroke patients, extracting cerebral artery stenosis features. A random forest model achieved 93.6% accuracy in predicting upper limb function recovery, with stenosis in the internal carotid artery origin and C1 segment being the most important predictors.

#### 3.4.2 Clinical feature prediction

##### 3.4.2.1 Prediction based on clinical data

Clinical data, including demographic characteristics, laboratory test results, physiological indicators, and microbiomic data, are utilized to predict the therapeutic efficacy of acupuncture. In stroke recovery, Yang et al. (101) used a classification and regression tree (CART) integrating clinical and lab indicators (age, onset time, Traditional Chinese Medicine syndrome type, hemoglobin, homocysteine) to predict acupuncture efficacy with 72.8% accuracy. For Bell’s palsy, Xiao et al. (102) found younger age, lower BMI, acute phase, and lower initial facial nerve grade predicted better recovery. In a large-scale study involving 745 patients with functional dyspepsia, Yin et al. (103) successfully predicted treatment response, symptom improvement, and other outcomes using only 28 routine clinical features, with the support vector machine (SVM) model showing stable performance. Baeumler et al. (104) showed chronic pain patients with higher temporal summation were 4.3 times more likely to benefit from acupuncture, suggesting central sensitization as a biomarker. Clinical feature models also show promise in depression (105), opioid dependence (106), and ischemic stroke (107).

##### 3.4.2.2 Prediction based on clinical response

*Deqi* specifically refers to a sensation experienced during acupuncture, characterized mainly by feelings of soreness, numbness, heaviness, and distension in the patient, while the physician feels a sense of tightness, tension, roughness, or stagnation under the needle. Achieving *deqi* is a crucial aspect of acupuncture, believed to be one of the key elements in producing therapeutic effects. Modern studies also suggest that achieving *deqi* leads to better treatment outcomes (108–110). In a study by Chen et al. (111) the characteristics of the *deqi* sensation reported by patients during their first acupuncture treatment, such as its presence, duration, intensity of distension, and intensity of pain, were used as inputs to a support vector machine (SVM) model to predict the overall treatment response for functional dyspepsia. The results showed that the model could distinguish between high responders and low responders, with an accuracy rate of 84%, confirming the clinical predictive value of the *deqi* sensation. The specific details are presented in Table 6.

**Table 6:** Detailed acupuncture efficacy prediction.

Author	Disease	Core method	Predictive marker
Liu et al. (98)	Migraine	HCA, MVPA, SVM	Clinical, Neuroimaging features
Yang et al. (99)	Migraine	LR, Linear SVM	Neuroimaging features
Yin et al. (87)	Migraine	MVPA, SVC, SVR	Neuroimaging features
Gao et al. (88)	Neck pain	SVM, LR, KNN	Neuroimaging features
Baeumler et al. (104)	Chronic pain	LR	Clinical features
Tu et al. (95)	Chronic low back pain	SVR	Neuroimaging features
Wei et al. (89)	Chronic sciatica	SVR	Neuroimaging features
Yu et al. (96)	Primary dysmenorrhea	MVPA, SVR	Neuroimaging features
Zhan et al. (107)	Ischemic stroke	RF, XGBoost, SVM, LR,	Clinical features
Tang et al. (100)	Upper limb dysfunction after ischemic stroke	YOLOX, Faster R-CNN, TOOD, RF	Neuroimaging features
Yang et al. (101)	Post stroke recovery phase	DT	Clinical features
Xiao et al. (102)	Bell’s palsy	LR	Clinical features
Yang et al. (105)	Depression	BP neural network	Clinical features
Dong et al. (106)	Opioid dependence	DT	Clinical features
Chen et al. (111)	Functional dyspepsia	SVM	Clinical features
Yin et al. (97)	Functional dyspepsia	SVM	Neuroimaging features
Yin et al. (103)	Functional dyspepsia	SVM	Clinical features

SVM, Support Vector Machine; SVR, Support Vector Regression; SVC, Support Vector Classifier; LR, Logistic Regression; RF, Random Forest; XGBoost, Extreme Gradient Boosting; KNN, K-Nearest Neighbors; MVPA, Multivariate Pattern Analysis; HCA, Hierarchical Clustering Analysis; DT, Decision Tree; LASSO, Least Absolute Shrinkage and Selection Operator.

#### 3.4.3 Summary

This section discusses predictive indicators for acupuncture efficacy. The above findings indicate that combining pre-treatment neuroimaging and clinical data with AI algorithms can effectively predict individual treatment responses. Spontaneous brain activity and brain network functional connectivity are closely associated with therapeutic outcomes, while clinical features such as demographic information, medical history, and the microbiome also demonstrate strong predictive potential. However, challenges remain. First, data quality and standardization are major concerns, as most studies rely on small, single-center samples, limiting model generalizability. Second, current predictions are typically based on pre-treatment baseline data, yet acupuncture involves long-term regulatory processes. To address these issues, three recommendations are proposed: (1) conduct large-scale multicenter studies and establish standardized data sharing platforms to validate and optimize predictive models; (2) develop dynamic predictive models that integrate baseline and longitudinal data to enable real-time efficacy evaluation and adaptive treatment adjustments; and (3) explore pathways for clinical translation of predictive results. The ultimate goal is to develop a clinical prediction-based diagnostic and treatment system that identifies acupuncture-sensitive patients through clinical data, recommends personalized acupoint combinations based on symptom profiles, and helps formulate optimal treatment plans.

## 4 Conclusion

This paper provides a review of the current research status of AI technology in the clinical field of acupuncture, covering four key areas: acupoint selection, localization, improving acupuncture safety, and predicting acupuncture efficacy. In the area of acupoint selection, the Apriori algorithm and clustering analysis are undoubtedly the most commonly used AI algorithms. Artificial neural networks (ANNs) have also been applied to clinical acupoint selection. In acupuncture point localization research, indirect localization based on keypoint detection and direct localization using model-based algorithms are the main methods. Research has primarily focused on the localization of points on the head, face, and hands. Regarding acupuncture safety, AI applications have mainly focused on needle management and treatment procedures. Needle management has transitioned from manual counting to intelligent visual recognition, though further clinical trials are needed to validate its application in treatment procedures. In the prediction of acupuncture efficacy, the primary focus has been on the objective prediction of treatment outcomes using neuroimaging features and clinical characteristics. Various machine learning and deep learning technologies, including support vector machines, decision trees, logistic regression, random forest, and object detection models, have been applied, achieving high prediction accuracy. Despite ongoing technological advancements, the transition of AI-assisted acupuncture from research to clinical application still faces multiple challenges. First, the interpretability of deep learning models remains a critical issue—clinicians need to understand the underlying logic by which AI identifies acupoints or predicts therapeutic effects, in order to build trust and ensure diagnostic transparency. Second, the integration of AI with traditional clinical workflows must be optimized to avoid increasing practitioners’ workload. Furthermore, addressing regulatory concerns and establishing standardized protocols for AI-assisted interventions are essential for mitigating legal risks and ensuring patient safety across diverse clinical settings. The ultimate goal is for AI to enhance the precision of traditional theories without altering the personalized nature of acupuncture treatment.
